# Lung microdialysis and *in vivo* PK/PD integration of cefquinome against *Actinobacillus pleuropneumoniae* in a porcine experimental lung infection model

**DOI:** 10.3389/fvets.2024.1390336

**Published:** 2024-03-26

**Authors:** Yuqin Chen, Min Li, Dehai Su, Shiyu Xiong, Youshu Feng, Qin Deng, Huanzhong Ding

**Affiliations:** Guangdong Key Laboratory for Veterinary Drug Development and Safety Evaluation, College of Veterinary Medicine, South China Agricultural University, Guangzhou, China

**Keywords:** cefquinome, *Actinobacillus pleuropneumoniae*, microdialysis, PK/PD integration model, porcine pleuropneumonia

## Abstract

This study aim to explore the application of microdialysis in pharmacokinetic (PK) and pharmacodynamic (PD) integration of cefquinome against *Actinobacillus pleuropneumoniae* in a porcine experimental lung infection model. The model was established via intratracheal inoculation where average bacterial counts (CFU) in the lungs of infected pigs reached 6.57 log_10_ CFU/g after 3 h. The PK profiles of unbound cefquinome in lung dialysates were determined following intramuscular injection of single doses of 0.125, 0.25, 0.5, 1, 2, and 4 mg/kg. Lung dialysate samples were collected using microdialysis at a flow rate of 1.5 μL/min until 24 h. The PD studies were conducted over 24 h based on 10 intermittent dosing regimens and total daily doses ranged from 0.25 to 4 mg/kg and dosage intervals included 12 and 24 h. The lung tissue was collected after 24 h of treatment and homogenized for bacterial counts. The relationships between PK/PD parameters derived from lung dialysates and drug efficacy were analyzed using an inhibitory sigmoid E_max_ model. The percentage of time the free drug concentration exceeded the minimum inhibitory concentration (%*f*T > MIC) was the PK/PD index best describing the antimicrobial activity (*R*^2^ = 0.96) in the porcine experimental infection model. The %*f*T > MIC values required to achieve net bacterial stasis, 1, 2 and 3 log_10_ CFU/g reductions in the lung were 22.45, 28.86, 37.62, and 56.46%, respectively. Cefquinome exhibited time-dependent characteristics against *A. pleuropneumoniae in vivo*. These results provide valuable insights into the application of microdialysis in PK/PD integration model studies and optima regimen of cefquinome for the treatment of porcine respiratory diseases caused by *A. pleuropneumoniae*.

## Introduction

*Actinobacillus pleuropneumoniae* is a pathogen in pigs that colonizes and proliferates in the lungs, trachea and bronchi, leading to severe edema, inflammation, hemorrhage and necrosis (1). Cefquinome has been approved for treating various livestock diseases, including respiratory diseases, septicemia, foot rot, and acute mastitis (2). Its efficacy against *Pasteurella multocida, Haemophilus parasuis, Klebsiella pneumoniae* and *A. pleuropneumoniae* has been demonstrated by low minimum inhibitory concentrations (MICs) observed in *in vitro* studies (3, 4). Nonetheless, the irrational and overuse of cefquinome in veterinary clinical practice may increase the risk of therapeutic failure and antimicrobial resistance, significantly shorten the drug’s service life.

Pharmacokinetic/pharmacodynamic (PK/PD) integration models are widely utilized to establish rational drug dosing regimens and to prevent the development of bacterial resistance (5). Because most bacterial lung infections occur in the pulmonary interstitial fluid rather than in plasma, using antibiotic dosing regimens guided by plasma PK data may not be optimal (6). Therefore, the U.S. Food and Drug Administration (FDA) advocates for monitoring free drug concentrations at the site of infection to ensure adequate levels, thereby preventing treatment failure and resistance development (7). However, for infections occurring in deep-seated tissues or organs such as the lungs, brain, and kidney, detecting drug concentrations therein still presents certain difficulties (8).

Microdialysis (MD), a probe-based sampling technique, has been effectively implemented for measuring levels of antibiotics, including imipenem (9), levofloxacin (10), tobramycin (11), cefpodoxime (12) and gatifloxacin (13) in pulmonary interstitial fluid. This approach fulfills the practical needs of pharmacological monitoring and PK/PD studies. As a unique tool, it enables the direct measurement of free drug concentrations in dialysate samples and in tissue interstitial and organs (14). Furthermore, microdialysis can be conducted in both awake and anesthetized animals, allowing for continuous sampling of lung interstitial fluid (15).

In this study, we attempted to investigate the feasibility of using microdialysis to measure the concentration of free cefquinome in the lungs of a porcine experimental lung infection model. Additionally, we examined the relationship between the *in vivo* antibacterial activity of cefquinome and the PK/PD parameters derived from the lungs of infected pigs. Finally, we assessed the PK/PD parameters necessary to achieve antibacterial effects against *A. pleuropneumoniae*. The obtained data deepen our understanding of cefquinome and provide valuable insights for clinical dosage decisions.

## Materials and methods

### Chemicals and reagents

Cefquinome sulfate standard (>97%) and Ringer’s solution were purchased from Yuanye Bio Technology (Shanghai, China). Cefquinome sulfate injection (25 mg/mL, Lot 907,009,027) was purchased from Qilu Animal Health Products (Jinan, China). Nicotinamide adenine dinucleotide (NAD) was provided by MYM Biological Technology (Beijing, China). Pentobarbital sodium was purchased from Jian Yang Biotechnology (Guangzhou, China). Procainamide hydrochloride was supplied by Harbin Longjiang Biotechnology (Harbin, China).

### Minimum inhibitory concentration determination

The *A. pleuropneumoniae* standard (CVCC259) was obtained from the Chinese Veterinary Microorganism Culture Collection Center (Beijing, China) and used for these experiments. The bacteria were cultivated in Mueller-Hinton Agar (MHA) and Tryptic Soy Broth (TSB) (Guangdong Huankai Microbial Technology, Guangzhou, China) supplemented with sterile newborn bovine serum (4%, V/V; Guangzhou Ruite Biotechnology, Guangzhou, China) and NAD (10 mg/L). After incubation in a constant temperature shaker at 37°C and 200 rpm/min for 8 h in TSB, logarithmic phase bacteria were diluted and the final concentration of 5 × 10^5^ colony forming units (CFU)/mL was applied to test the minimum inhibitory concentration (MIC) using the microdilution method (Clinical and Laboratory Standards Institute, CLSI) (16).

### Establishment of the porcine experimental lung infection model

Crossbred pigs (Landrace × Large White × Duroc) 6 weeks of age weighting of 13.5–15.0 kg were obtained from Guangdong Jiajing Swine Farm (Guangdong, China). These animals were housed at the Laboratory Animal Center of South China Agricultural University, where they had access to freshwater *ad libitum* and were fed antibiotic-free diets twice daily. After a 7-day acclimatization period, clinically asymptomatic pigs were selected for subsequent experiments. All experimental procedures were conducted in accordance with the requirements of the Committee on the Ethics of Animals of South China Agricultural University (Approval number: 2021A013).

These pigs lacking clinical symptoms were inoculated with diluted exponential growth phase cultures of *A. pleuropneumoniae* (diluted in sterile saline to approximately 3.5 × 10^7^ CFU/mL) at a dosage of 0.2 mL/kg administered intratracheally. At 3 h post-inoculation, the pigs exhibited typical clinical symptoms compared to healthy pigs, confirming the successful establishment of the porcine lung infection model.

### Implantation of microdialysis probe

Thirty minutes before surgery, the infected pigs were placed under general anesthesia induced by pentobarbital sodium and local anesthesia by the injection of procainamide hydrochloride. The animals were immobilized in a prone position using a restraint frame. The chest hair was shaved and the skin was disinfected prior to surgery. A skin incision of 4–6 cm in length was made on the left side of the pig’s chest and blunt dissection of the subcutaneous tissue was performed to better expose the intercostal muscles. Subsequently, a split tube with a steel introducer was inserted vertically through the intercostal muscle layer between the 9th and 10th ribs into the lung parenchyma. After the steel introducer was replaced by a CMA 20 microdialysis probe (CMA Microdialysis AB, Kista, Sweden), split tube was then removed. The incision surface was then sealed with tissue adhesive and the probe was secured in the muscle layer using surgical sutures to prevent displacement. Following implantation, the probe was connected to a BASi infusion pump (West Lafayette, IN, United States) and Ringer’s solution was continuously infused at a rate of 1.5 μL/min until the pigs regained consciousness.

### Calibration of microdialysis probe

The *in vitro* relative recovery (RR) was tested by dialysis and retrodialysis. Cefquinome in Ringer’s solution were used for perfusion at 50, 100 and 500 ng/mL at flow rates of 0.5, 1.0, 1.5 and 2.0 μL/min. The relative recovery by dialysis (RR_dialysis_) and retrodialysis (RR_retrodialysis_) were calculated using equations (1, 2), as detailed in the study by Zhang et al. (17).


(1) $$RR_{\mathrm{dialysis}}\left(\%\right)=\left(C_{\mathrm{dial}}/C_{ext}\right)\times 100$$

(2) $$RR_{\mathrm{retrodialysis}}\left(\%\right)=\left(\left(C_{\mathrm{perf}}-C_{\mathrm{dial}}\right)/C_{\mathrm{perf}}\right)\times 100$$

where C_dial_ is the concentration of cefquinome in the dialysate, C_ext_ is the concentration of cefquinome in the Ringer’s solution around the microdialysis probe, and C_perf_ is the concentration of cefquinome in the perfusate.

The *in vivo* RR was tested by retrodialysis using Ringer’s solution containing cefquinome at 500 ng/mL at a perfusion flow rate of 1.5 μL/min as described (17).

### Experimental design and sample collection

PK Study: Following implantation of the microdialysis probe in the infected animals, a minimum of 45 min of microdialysis equilibration was conducted. The infected pigs were intramuscularly injected with cefquinome at doses of 0.125, 0.25, 0.5, 1, 2, and 4 mg/kg. Lung dialysate samples were collected at 0, 0.15, 0.25, 0.5, 0.75, 1, 2, 4, 6, 8, 12 and 24 h using a flow rate of 1.5 μL/min and each collection lasting for 15 min. Lung dialysate samples were stored at −80°C until analysis. Eight pigs were randomly assigned to each dose group.

PD Study: Infected pigs were treated with cefquinome at five total doses (0.25, 0.5, 1, 2, and 4 mg/kg) administered by intramuscular injection, divided into two dosing intervals (every 12 and 24 h) over the 24 h period. Three pigs were allocated to each dosing regimen. After 24 h of drug administration, the pigs were sacrificed and the lungs were immediately removed for CFU determinations. Untreated control group pigs (treated with 0.9% sterile physiological saline solution) were also sacrificed at 0 and 24 h and their lungs were immediately removed for bacterial testing as per above.

Under aseptic conditions, 5 g lung tissue samples were collected from three different areas of infected lungs, homogenized and combined and 1 g of the homogenized mixtures were serially diluted 10-fold with pre-cooled 0.9% sterile physiological solution for CFU determinations. Subsequently, 20 μL of the dilutions were plated on MHA plates and incubated for 24 h. Bacterial counting was conducted three times in the same lung with the count of *A. pleuropneumoniae* expressed as the mean value as CFU/g.

### Determination of cefquinome concentrations in lung dialysate

The concentration of cefquinome in dialysate sample was analyzed using an Agilent 1,200 series high-performance liquid chromatography (HPLC) unit coupled with an API 4000 triple quadrupole mass spectrometer equipped with an electrospray ionization source (Agilent Technologies, Santa Clara, CA, United States) as previously described (17) with slight modifications.

The lung dialysate samples (20 μL) were mixed with 80 μL of ultrapure water and directly analyzed. The standard curve (*R*^2^ > 0.99) was established using seven calibration standards of cefquinome in the Ringer’s solution, ranging from 2 to 500 ng/mL.

### Data analysis

The PK parameters for each dosage regimen were calculated using compartmental models in WinNonlin software, version 5.2.1 (Pharsight, CA, United States). The PK/PD indices, including the maximum concentration of free cefquinome (*f*C_max_), the area under the concentration-time curve over 24 h (*f*AUC_24h_), and the percentage of time over the first 24 h that the concentration of free cefquinome exceeded the minimum inhibitory concentration (%*f*T > MIC), were calculated using a non-compartmental model combined with MIC.

To explore the relationship between PK/PD parameters derived from lung dialysate and antimicrobial effects, an inhibitory sigmoid maximum effect model in WinNonlin software was employed and is described by the equation (3):


(3) $$E=E_{\text{max}}-\left(E_{\text{max}}-E_{0}\right)\times C_{e}^{N}/\left(C_{e}^{N}+EC_{50}^{N}\right)$$

where E represents the antimicrobial effect, defined as the change of bacterial count in the lung 24 h after administration. E_max_ represents the change of bacterial count in the control group lungs, E_0_ represents the maximum antibacterial effect after administered various cefquinome dosages during 24 h, C_e_ is the value of a certain PK/PD parameter (%*f*T > MIC, *f*AUC_24h_/MIC, *f*C_max_/MIC). EC_50_ is the PK/PD parameter value corresponding to a 50% reduction in maximum antibacterial effect and N is the Hill coefficient that describes the steepness of the curve.

## Results

### Susceptibility testing

The MIC of cefquinome against *A. pleuropneumoniae* CVCC 259 in TSB was 0.008 μg/mL.

### RRs of microdialysis

**Figure 1:**
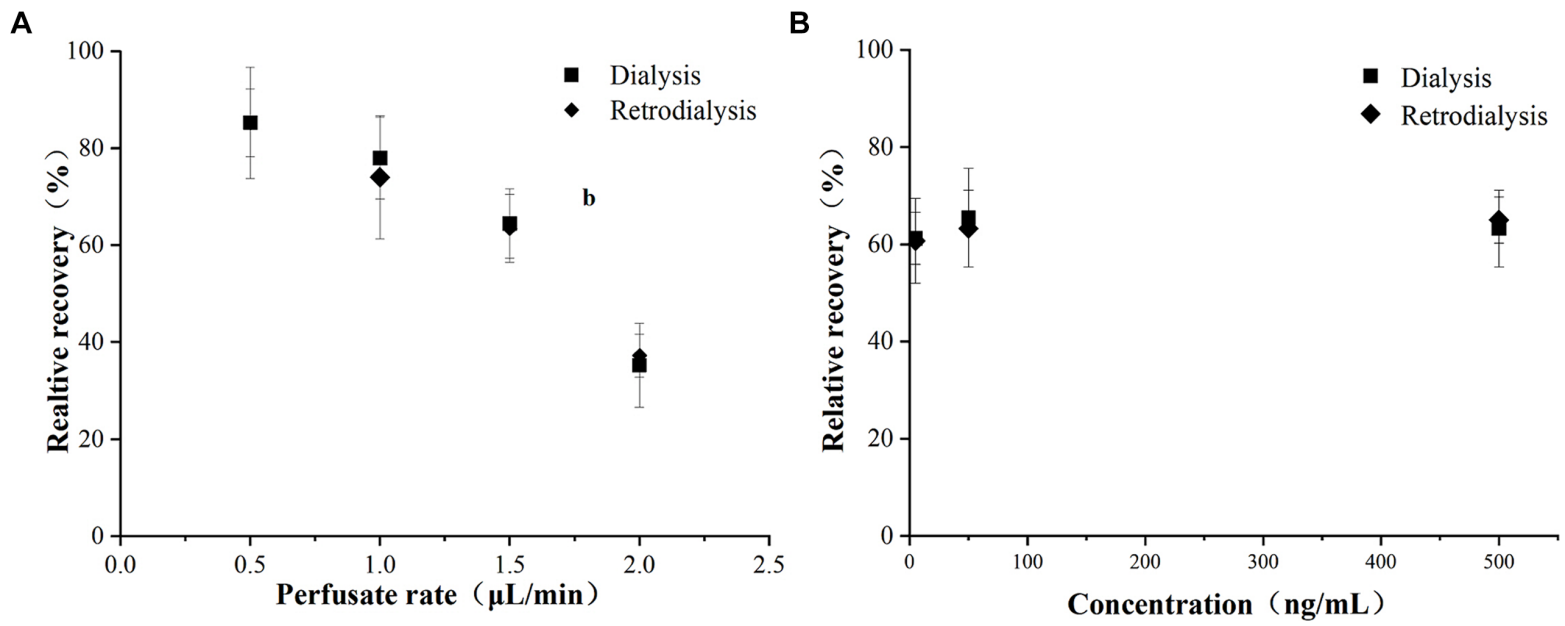
Influence of cefquinome **(A)** perfusion flow rate and **(B)** concentration on *in vitro* relative recoveries determined by dialysis and retrodialysis as indicated. Each symbol represents the mean value ± SD.

The *in vitro* RRs of the microdialysis probe determined by dialysis and retrodialysis are shown in Figure 1. The *in vitro* RRs for flow rates ranging from 0.5 to 2 μL/min for dialysis and retrodialysis decreased from 85.62 to 24.51% and from 83.27 to 33.61%, respectively. The RRs remained consistent as the concentration of cefquinome increased from 50 to 500 ng/mL. These findings suggest that determining the *in vivo* RRs could be determined by retrodialysis. Considering the superior RR and short interval, a flow rate of 1.5 μL/min (RRs: 59.51 and 64.32% for dialysis and retrodialysis, respectively) was selected for the following experiments.

**Figure 2:**
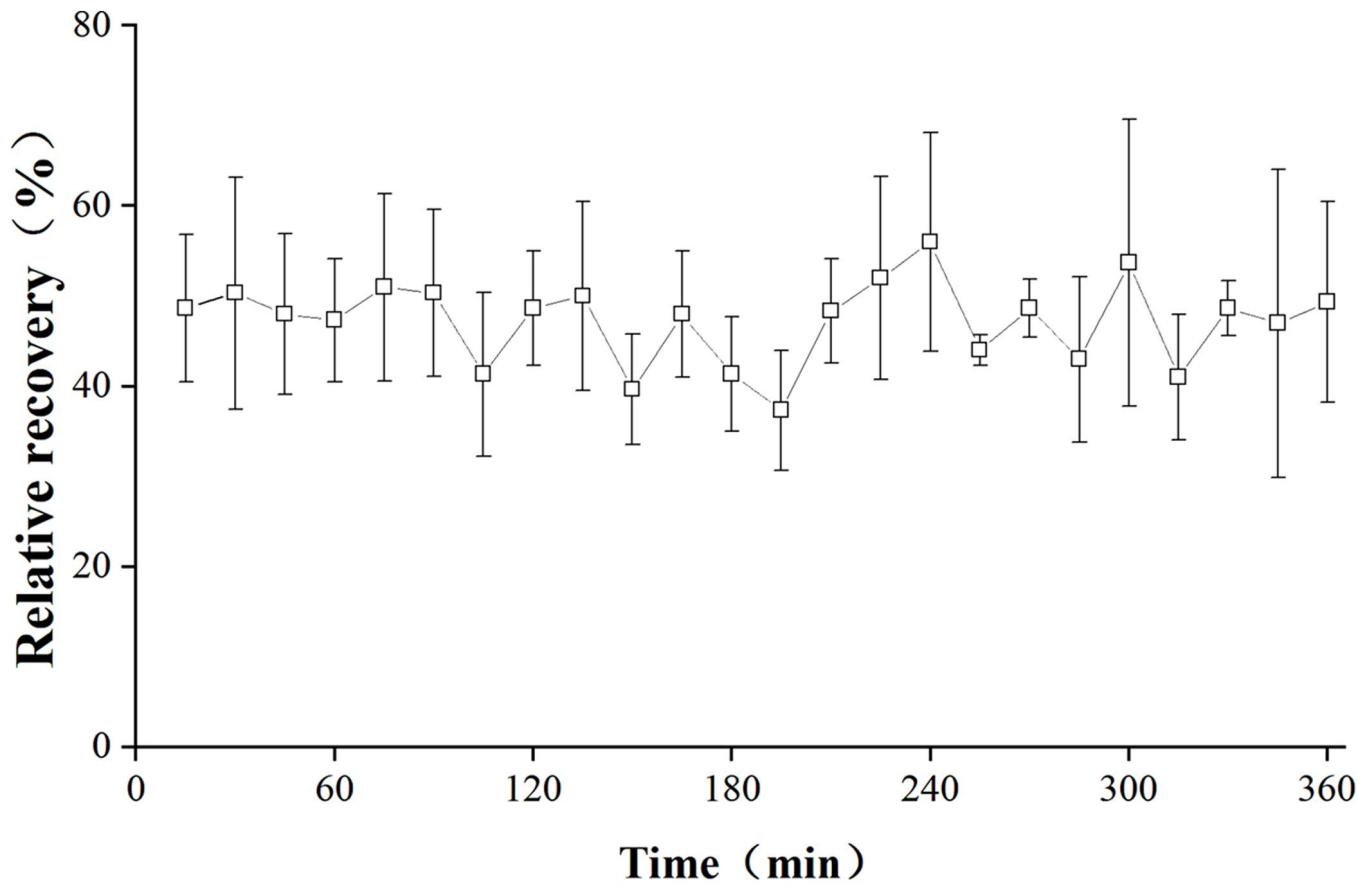
*In vivo* RRs of cefquinome in the lung of infected pigs. Each symbol represents the mean value ± SD.

The *in vivo* RRs of the probe are depicted in Figure 2. The RRs remained stable over a 6-h period, with mean values of 47.23%.

### Porcine experimental infection lung infection model

The successful establishment of the experimental lung infection model relied primarily on the observation of clinical symptoms and bacteriological examination (CFU counts). Infected pigs exhibited decreased appetite, depression, elevated body temperature and coughing within 3 h. In untreated control pigs, the initial bacterial population (3 h after infection) in the lung ranged from 5.97 to 6.88 log_10_ CFU/g and increased by 2.37 log_10_ CFU/g after 24 h.

### Pharmacokinetics of free cefquinome in the lung

**Figure 3:**
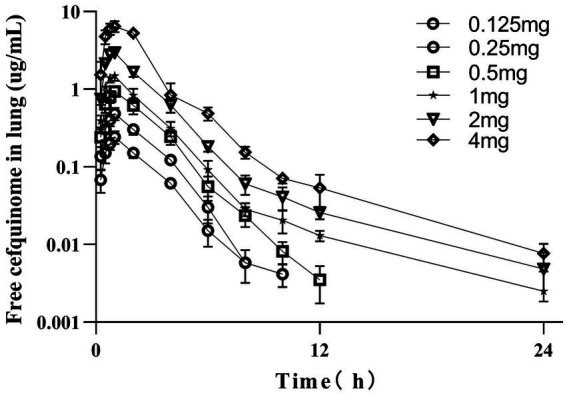
PK profile of cefquinome in lungs of infected pigs after a single intramuscular dose. Each symbol represents the mean value ± SD.

The concentrations of free cefquinome in lungs of infected pigs following single intramuscular doses of 0.125, 0.25, 0.5, 1, 2, and 4 mg/kg are depicted in Figure 3. The PKs were best described by a two-compartment model with first-order absorption for the 0.5, 1, 2, and 4 mg/kg dose groups, while a one-compartment model with first-order absorption was most suitable for the 0.125 and 0.25 mg/kg dose groups. These concentrations were utilized to calculate various PK parameters listed in Table 1. Over the dose range studied, kinetics remained linear, with both C_max_ and AUC exhibiting linear kinetics at these doses.

**Table 1:** PK parameters of free cefquinome following single intramuscular doses in a porcine experimental lung infection model.

Dose (mg/kg)	T_max_ (h)	C_max_ (μg/mL)	T_1/2ke_ (h)	T_1/2α_ (h)	T_1/2β_ (h)	AUC (μg h/g)
0.125	0.83 ± 0.27	0.25 ± 0.05	1.32 ± 0.22	–	–	0.72 ± 0.24
0.25	1.03 ± 0.13	0.40 ± 0.06	1.00 ± 0.18	–	–	1.11 ± 0.18
0.5	0.89 ± 0.21	0.68 ± 0.13	–	0.92 ± 0.20	4.50 ± 0.74	2.07 ± 0.40
1	0.95 ± 0.14	1.37 ± 0.29	–	0.94 ± 0.32	5.54 ± 1.32	3.77 ± 0.82
2	0.82 ± 0.12	2.76 ± 0.91	–	0.94 ± 0.25	4.81 ± 1.68	7.26 ± 1.24
4	0.96 ± 0.14	6.14 ± 1.03	–	1.03 ± 0.16	5.52 ± 1.05	16.04 ± 2.57

T_max_, time to maximum concentration; C_max_, maximum concentration during the dosage interval; T_1/2ke_, elimination the half-life in one-compartmental open model; T_1/2ɑ,_ distribution half-life in two-compartmental model; T_1/2β,_ elimination half-life in two-compartmental model; AUC, area under the concentration-time curve after each administration. The values shown are mean ± SD.

### Dose-fractionation studies

**Figure 4:**
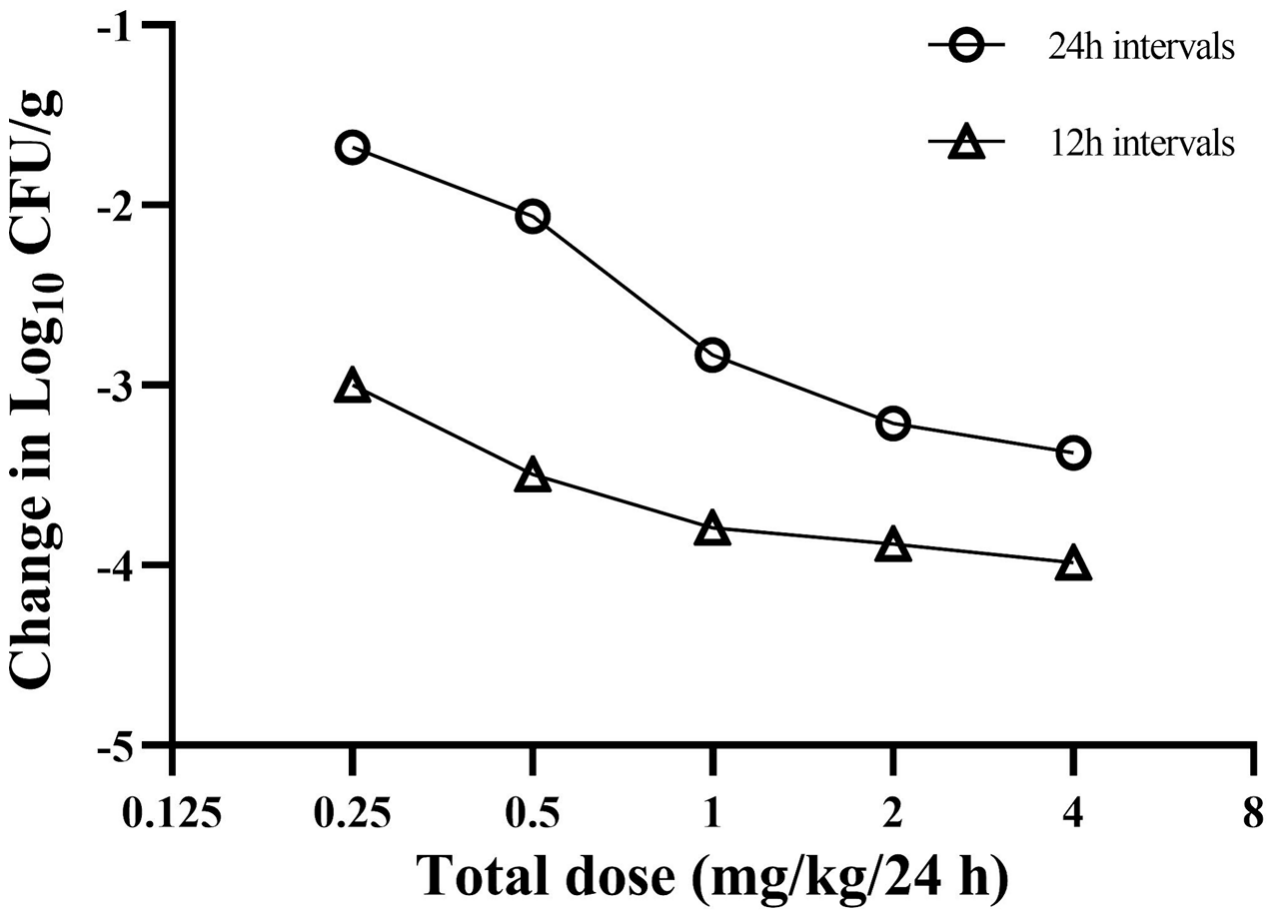
Relationship between cefquinome dosing interval and efficacy against *Actinobacillus pleuropneumoniae* in a porcine experimental lung infection model. Each symbol represents the mean data from three infected pigs.

The dose–response relationships for *A. pleuropneumoniae* are presented in Figure 4. With the escalation in the total dose, the bacterial reduction in the lungs altered from 1.62 to 3.15 log_10_ CFU/g in the group with 24-h dosing intervals, and from 2.96 to 3.80 log_10_ CFU/g in the group with 12-h dosing intervals. Across all studied dosing regimens, the 12-h interval dosing regimen exhibited superior bacterial eradication of *A. pleuropneumoniae* during a 24-h treatment period compared to the 24-h interval dosing regimen. This seems to suggest that the amplification of the total dosage and the reduction of the time interval between doses may enhance the effectiveness in the treatment of the disease investigated in this study.

### Magnitude of the PK/PD index associated with efficacy

**Figure 5:**
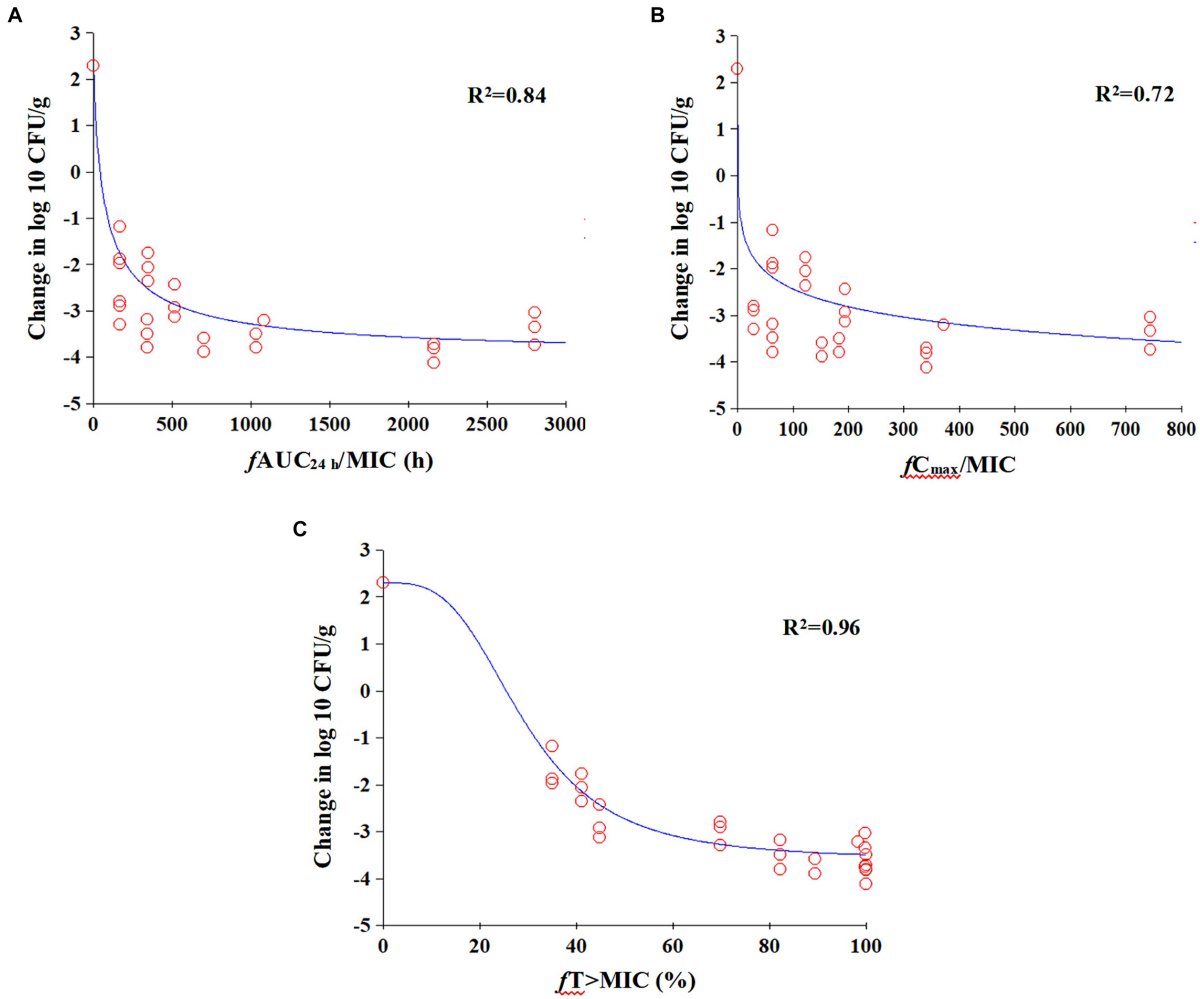
E_max_ relationships for the three PK/PD parameters versus antimicrobial effect. **(A)** *f*AUC_24h_/MIC; **(B)** *f*C_max_/MIC; **(C)** *f*T% > MIC.

The relationships between antibacterial efficacy and PK/PD indices (*f*AUC_24h_/MIC, *f*C_max_/MIC, %*f*T > MIC) derived from lung are shown in Figure 5. The results shown that %*f*T > MIC (R^2^ = 0.96) was the optimal PK/PD index correlated to efficacy, compared with *f*AUC_24h_/MIC (R^2^ = 0.84) and *f*C_max_/MIC (R^2^ = 0.72). We also calculated EC_50_, N, E_0_, and E_max_, and estimated the %*f*T > MIC values required to achieve different antimicrobial effects, as listed in Table 2. The estimated %*f*T > MIC values required for net bacterial stasis, 1, 2 and 3 log_10_ CFU/g reduction in the lung at 24 h were 22.45, 28.86, 37.62, and 56.46%, respectively.

**Table 2:** The values of PK/PD parameters and %*f*T > MIC required to achieve various degrees of antibacterial efficacy in the porcine lung infection model.

Parameter	Values
E_max_ (△Log_10_ CFU/g)	2.28
E_0_ (△Log_10_ CFU/g)	−3.82
EC_50_ (%)	27
Slope (N)	2.64
%*f*T > MIC for net static effect during 24 h (%)	22.45
%*f*T > MIC for 1-Log_10_ CFU/g reduction during 24 h (%)	28.86
%*f*T > MIC for 2-Log_10_ CFU/g reduction during 24 h (%)	37.62
%*f*T > MIC for 3-Log_10_ CFU/g reduction during 24 h (%)	56.46

%*f*T > MIC, the percentage of time that free drug concentration in lung remains above the MIC. E_0_, the maximum effect after administered various cefquinome dosages during 24 h. E_max_, the change of bacterial count in control group. EC_50_, the %*f*T > MIC values required to achieve 50% of the maximal antibacterial effect during 24 h. N, the Hill coefficient which describes the steepness of the %*f*T > MIC and effect curve.

## Discussion

For respiratory infections, poor penetration at the target site and consequent insufficient antibiotic exposure to the pathogen may lead to treatment failure (18). The measurement of drug concentrations in the lungs remains challenging due to their protected anatomical location and heightened vulnerability (15). The traditional approaches such as bronchoalveolar lavage (BAL) and whole lung tissue homogenates, employed to study lung tissue antimicrobial levels and have inherent limitations. Whole lung tissue homogenates require the consumption of a large number of experimental animals for PK studies (19). BAL alters the composition of the epithelial lining fluid and cannot provide sampling at all necessary time points from single animals (20, 21). Additionally, due to the uncertainty of the dilution factor between bronchoalveolar lavage fluid and the actual interstitial or epithelial lining fluid concentrations within the lungs, PK characteristics are often described based on total drug content rather than the free drug concentration (22–24). Microdialysis has been applied to measure the concentration of antimicrobial drugs in the interstitial fluid and provide preclinical drug development and clinical PK/PD data for antimicrobial drugs (25, 26). Therefore, this study employed microdialysis to investigate the PK characteristics of unbound cefquinome in the lung and evaluate the *in vivo* antibacterial effect of cefquinome against *A. pleuropneumoniae*.

Porcine pleuropneumonia can manifest in various clinical forms, among which the acute form characterized by fibrino-haemorrhagic and necrotizing pleuropneumonia is usually fatal and results in significant economic losses (27, 28). Thus, we adopted intratracheal inoculation of *A. pleuropneumoniae* to induce acute pneumonia in pigs for PD evaluation. At 3 h post-inoculation, the pigs exhibited noticeable respiratory symptoms and the mean bacterial burden in the lungs reached approximately 6.57 log_10_ CFU/g, subsequently increasing by 2.37 log_10_ CFU/g units over the next 24 h. These results indicated that intratracheal inoculation can effectively replicate acute pneumonia caused by *A. pleuropneumoniae*.

We examined the PK profile of unbound cefquinome in the lungs of infected pigs. Consistent with previous plasma PK studies in piglets (29), the PK characteristics of free cefquinome concentrations in the lungs of infected pigs receiving intramuscular injections of doses at 0.5, 1, 2, and 4 mg were best described by a first-order absorption two-compartment open model. Nonetheless, the data from the low-dose group (0.125 and 0.25 mg groups) indicate that lung free concentrations fit a first-order absorption one-compartment open model. The reason for this outcome may be the short half-life of cefquinome, resulting in the free cefquinome not being detectable in lung dialysate at 12 h post-administration in the 0.125 mg and 0.25 mg dose groups, respectively. Furthermore, following intramuscular administration of 2 mg/kg cefquinome, we observed that the C_max_ of cefquinome in the lungs of infected pigs was lower than the corresponding value previously reported in piglet plasma (4.01 μg/mL) (29). Due to the lung-blood barrier, most antimicrobial agents cannot achieve complete equilibrium between lung and plasma concentrations. Using the free concentrations in lung rather than total concentrations in plasma may be more predictive of *in vivo* antimicrobial efficacy.

Cefquinome has shown time-dependent antimicrobial activity against both Gram-negative and Gram-positive bacteria in *in vivo* studies and the %*f*T > MIC values are often used to predict antibacterial efficacy. For example, a PD study involving cefquinome against *Staphylococcus aureus* strain ATCC 29213 using a neutropenic murine thigh infection model revealed that the %*f*T > MIC values required for bacteriostasis, 0.5 and 1 log_10_ CFU/thigh reduction were 31.61, 38.48 and 54.01%, respectively (30). Likewise, another study evaluated the activity of cefquinome against *Escherichia coli* in a neutropenic murine thigh infection model, determined that the %*f*T > MIC values required for bacteriostasis, 1 and 2 log_10_ CFU/thigh reductions were 28.01, 37.23 and 51.69%, respectively (31). Our results demonstrated that %*f*T > MIC (R^2^ = 0.97) had the highest correlation with efficacy in comparison to *f*AUC/MIC (R^2^ = 0.87) and *f*C_max_/MIC (R^2^ = 0.79), demonstrating its time-dependent characteristic.

Based on results using dose fractionation methodology, we observed a corresponding enhancement in bacterial reduction in the lungs with an increase in the total amount of drug for both single and fractionated dosing regimens over 24 h of treatment. Among the five total doses studied, a superior antimicrobial efficacy in the lung was observed when administering the total dose in divided fractions rather than as a single administration. Similar findings were reported in the study of cefprozil against *Haemophilus influenzae* where once-daily administration was not as effective as twice-daily dosing (32). Adjusting the dosing schedule such as implementing multiple daily doses, may provide a more attractive strategy for improving clinical cure rates.

Using an inhibitory Sigmoid E_max_ model, the %*f*T > MIC values required for achieving bacterial growth inhibition as well as 1, 2 and 3 log_10_ CFU/g reductions in the lung of infected pigs were 22.45, 28.86, 37.62 and 56.46%, respectively. The antimicrobial efficacy of cefquinome against *A. pleuropneumoniae*, as assessed using a neutropenic murine thigh infection model, showed that the %*f*T > MIC values required for achieving bacterial stasis and 1 and 2 log_10_ CFU reductions were 31.61, 38.48 and 54.01%, respectively (33). In the neutropenic murine thigh infection model, the %*f*T > MIC values required to achieve the same antimicrobial efficacy are higher than those in the porcine experimental lung infection model. This disparity could be attributed to the growth phases of *A. pleuropneumoniae* in murine thigh and pig lung tissue. Evidence suggests that cephalosporins exert bacteriostatic effects by inhibiting cell wall synthesis and demonstrate stronger bactericidal activity against actively growing bacteria (34, 35). Therefore, it is possible that *A. pleuropneumoniae* may exhibit different antibiotic sensitivities in pig lung tissue compared to murine thigh tissue. Additionally, mice were injected with cyclophosphamide to establish the neutropenic murine infection model, which may decrease the clearance ability of the animals. Consequently, the porcine experimental lung infection model developed in this study simulates the internal environment of target tissues under clinical infection conditions and provides valuable insights for guiding clinical drug administration.

In this study, we successfully characterized the PK/PD characteristics of cefquinome against *A. pleuropneumoniae in vivo* using microdialysis and %*f*T > MIC was the PK/PD parameter most strongly correlated with antibacterial efficacy. However, this study had several limitations. Implementing a multi-interval dosing strategy can substantially enhance %*f*T > MIC values, thereby ensuring the optimal efficacy of time-dependent antibiotics in the treatment of bacterial infections. For large animals like cattle, sheep, horses, and pigs, shorter dosing intervals (less than 12 h) may present practical and financial challenges (36). Therefore, cefquinome was administered only at 12 and 24 h intervals for the treatment of *A. pleuropneumoniae* infection in pigs for this PD study. Additionally, only the standard strain CVCC259 was used as the reference strain to establish the infection model. To develop optimized treatment strategies for clinical application, further drug treatment studies should encompass a broader range of serotypes and varying resistance sensitivities of *A. pleuropneumoniae*.

## Data availability statement

The original contributions presented in the study are included in the article/supplementary material, further inquiries can be directed to the corresponding author/s.

## Ethics statement

The animal study was reviewed and approved by the Committee on the Ethics of Animals of South China Agricultural University.

## Author contributions

YC: Writing – original draft, Writing – review & editing. ML: Writing – original draft. DS: Writing – original draft. SX: Writing – review & editing. YF: Writing – review & editing. QD: Writing – review & editing. HD: Writing – review & editing, Conceptualization, Funding acquisition, Methodology, Resources, Supervision.
